# Forgiveness as a Mediator between Psychological *Suzhi* and Prosocial Behavior in Chinese Adolescents

**DOI:** 10.3390/bs12090330

**Published:** 2022-09-11

**Authors:** Xu Chen, Hongxia Zhao, Dajun Zhang

**Affiliations:** 1 Normal College, Jimei University, Xiamen 361021, China; 2 Psychology Department, Southwest University, Chongqing 400715, China

**Keywords:** psychological *suzhi*, forgiveness, prosocial behavior, Chinese adolescent

## Abstract

Prosocial behavior contributes to the well-being of individuals as well as the harmonious development of society. This research aimed to reveal the mechanisms underlying the relationship between the psychological *suzhi* and prosocial behavior of Chinese adolescents with the consideration of time. A total of 477 adolescents (228 boys, 49.1%; M_age_ = 14.04 and SD = 1.77) from southwest China completed three questionnaires during waves 1 and 2. Results showed that psychological *suzhi* and forgiveness were positively correlated with prosocial behavior in both waves 1 and 2; psychological *suzhi* significantly predicted both current and three months later prosocial behavior and forgiveness played a mediating role in both immediate and lasting effects of psychological *suzhi* on prosocial behavior. Psychological *suzhi* and forgiveness are vital predictors of adolescents’ prosocial behaviors in China. Interventions based on psychological *suzhi* and forgiveness are essential to promote the development of pro-social behaviors.

## 1. Introduction

Prosocial behavior refers to the positive behaviors that meet social expectations and benefit others and society, which include sharing, comfort, donation, and voluntary services [1,2,3,4]. Prosocial behavior enhances not only an individual’s happiness and relationships with other members of society but also the development step of humanity in general [2]. Prosocial behavior in children is largely influenced by social norms, orientations, and internalization of culture-specific conventions [5,6,7]. Recent cross-cultural research has confirmed that prosocial behavior differs between cultures, and in China, a highly collectivized urban society, the behavioral development of children is particularly unique [1]. Psychological *suzhi* is a concept local to China and is also the focus of current educational departments. Numerous studies have demonstrated that psychological *suzhi* is inseparable from the development of children’s behavior [8,9,10,11,12]. However, research on psychological *suzhi* and prosocial behavior remains limited, especially concerning longitudinal studies. This has prevented researchers and educators from understanding the underlying relationship between psychological *suzhi* and prosocial behavior and the internal mechanism between these concepts. When considering time, this study aimed to reveal the influence of psychological *suzhi* on adolescent prosocial behavior and the role of forgiveness in this process to enrich the findings in the psychological *suzhi* and provide guidance to educational practices for increasing prosocial behavior in adolescents.

### 1.1. Association between Psychological suzhi and Adolescent Prosocial Behavior

Psychological *suzhi* is an individual’s trait, defined by Chinese scholars based on the background of quality-oriented education in China [13,14,15,16]. The term *suzhi* was derived from the education reform policy of the Chinese government (see http://www.moe.gov.cn (accessed on 1 August 2002)), which is similar to quality but with a greater scope [14]. This concept has been confirmed by many studies as a positive aspect of the development of children and adolescents. It has been included in The Handbook of Positive Psychology in School (Second Edition) [8,10,11,12,17,18]. Psychological *suzhi* comprises three parts: cognition quality, individuality, and adaptability [15,19]. Among them, cognitive quality is the most fundamental aspect of mental quality, which refers to an individual’s cognition regarding objects, such as critical thinking, creativity, and meta-cognition. Individuality is the core aspect and refers to personality qualities, such as achievement motivation, self-esteem, self-control, and self-confidence. Finally, adaptability is a reliable indicator of the activity of the other two components during social interactions, which are associated with emotional and interpersonal skills [11,14,19]. 

Adolescent behavioral development is inseparable from psychological *suzhi*. Previous studies have found that individuals with high psychological *suzhi* are more likely to develop good behavioral habits [8,20]. In contrast, those with low psychological *suzhi* are at risk of developing psychological and behavioral problems, such as social anxiety, depressive symptoms, suicidal ideation, bullying, and problematic behavior [9,13,17,19,21,22]. As mentioned above, psychological *suzhi* is an essential aspect of positive psychology that promotes an individual’s mental health and positive behavior development [8,9,11]. Pan et al. discussed the mediating role played by psychological *suzhi* in attachment and prosocial behaviors in adolescents and found that psychological *suzhi* was not only significantly positively correlated with adolescent prosocial behavior but was also a predictor of prosocial behavior [11]. However, there is currently insufficient evidence for the relationship between psychological *suzhi* and adolescent prosocial behavior. Enriching findings in this field is crucial to promoting the occurrence and maintenance of prosocial behavior among adolescents. Therefore, the purpose of this study was to expand current findings in this area. Based on the definition of psychological *suzhi* and the findings of Pan et al., Luo et al., and Mo et al., one may infer that psychological *suzhi* has a positive impact on adolescent prosocial behavior [8,9]. Therefore, hypothesis 1 of this study was that psychological *suzhi* would positively predict the prosocial behavior of adolescents. 

### 1.2. Psychological suzhi, Forgiveness, and Prosocial Behavior in Adolescents

In general, psychological *suzhi* is a relatively stable psychological trait, and its influence on an individual is realized through other state variables, such as loneliness and security [23]. Current literature suggests that several critical mediating variables contribute to the effect of psychological *suzhi* on individual behavior [23,24,25]. Forgiveness is a process of motivational transformation in which an individual transforms negative emotions (e.g., anger and hatred) into sympathy and love for the offender [26,27,28]. However, forgiveness has specific cultural differences [29,30]. Collective culture attaches more importance to harmony between people. As stated in a Chinese proverb, ‘be lenient wherever it is possible’. In the context of Chinese culture, Chinese scholars said that forgiveness refers to the feeling that one can forgive others and feel at ease [31]. Forgiveness is divided into deep and shallow forgiveness. Deep forgiveness refers to a type of emotion where one can actively forgive others and feel at ease with oneself. In contrast, superficial forgiveness refers to forgiving others at the request of outsiders (e.g., teachers, parents, and friends) [31]. The different definitions of forgiveness suggest that Chinese scholars have paid more attention to distinguishing between the initiative and negativity of forgiveness; that is, the motivation for forgiveness.

The occurrence of forgiveness is affected by stable factors. Studies in samples of both Chinese and European adolescents show that those with both high self-esteem and positive emotion regulation are more likely to forgive others [32,33,34]. Furthermore, Research by Ross et al. found that personality is closely connected to forgiveness, neuroticism is related to self-forgiveness, and agreeableness is related to relationships with others [35]. Afterward, Walker’s research based on a sample group in the American Midwest supported and further enriched this research finding. He found that personality is indeed a predictor of forgiveness, with neuroticism in personality significantly negatively predicting self-forgiveness, while agreeableness positively and significantly indicating others’ forgiveness [36]. Although there has not been any research that has directly focused on the relationship between psychological *suzhi* and forgiveness, several studies have confirmed that psychological *suzhi* positively predicts an individual’s level of self-esteem, which includes personality characteristics [14,21,25]. Therefore, as a factor superior to self-esteem and personality, psychological quality is likely to be an important predictor of forgiveness. Furthermore, many studies have shown that forgiveness, as a positive psychological trait, may help to promote prosocial behaviors among adolescents [31,37,38]. This is because people who forgive easily are more inclined to sympathize with criminals and are willing to help others with difficulty to gain more happiness [31]. Existing studies in adolescents have confirmed that forgiveness predicts prosocial behavior, where higher levels of forgiveness result in more prosocial behaviors [37,38]. Overall, psychological *suzhi* and forgiveness are both important factors that affect prosocial behavior, and psychological *suzhi* can promote the occurrence of forgiveness. Therefore, we proposed hypothesis 2, that forgiveness will mediate the relationship between adolescents’ psychological *suzhi* and prosocial behavior. 

Psychological *suzhi* has shown to be a relatively stable variable that does not change easily over a short period and has a continuous effect on adolescent development [11,15,17]. A two-year longitudinal study by Nie et al. found that psychological *suzhi* continuously affects adolescents’ academic performance [17]. In addition, a study by Chen showed that psychological *suzhi* significantly predicts the trend of adolescent relational aggression within one year [39]. Another study by Pan et al. found that adolescents’ current maternal attachment predicted their prosocial behavior one year later through psychological *suzhi* six months later [11]. However, the mechanism underlying the effect of psychological *suzhi* on prosocial behavior remains unclear; moreover, it is unknown whether the immediate and long-term impacts on prosocial behavior differ. Clarifying these issues will allow a deeper understanding of the true relationship between psychological *suzhi* and adolescent prosocial behavior and provide effective guidance for educational interventions. 

### 1.3. The Current Study

In the context of Chinese collectivism, prosocial behavior has its uniqueness from other countries. Based on the concept of Chinese native psychology, this research explored the internal mechanism of how psychological *suzhi* affects adolescent prosocial behavior and the instantaneous and continuous impact on prosocial behavior. We proposed a mediation model according to the literature, which suggests that psychological *suzhi* predicts adolescent prosocial behavior through forgiveness and verifies the model in the time dimension using a longitudinal design. We then tested this model and discussed its significance with reference to anti-bullying.

## 2. Methods

### 2.1. Participants

Adolescents in grades 7, 8, 10, and 11 from two middle schools in southwestern China completed this survey. All students voluntarily participated in the research. Firstly, we contacted the principals and head teachers of eligible schools to obtain their permission and select classes. Secondly, we introduced the survey and implementation time to the students in the class, and they were free to choose whether or not to participate. Finally, after obtaining the informed consent of the students, we used a paper questionnaire at the appointed time for measurement. The survey was divided into two waves: the first wave was conducted at the beginning of the spring semester, and the second wave was conducted at the end of the semester. There were 511 students who completed the survey for the first time, 478 for the second time, and 33 who dropped out after the first wave. Following analysis, the participant loss rate was approximately 6%, and the participant loss was random (*t* = 1.36 and *p* > 0.05). Students who participated in both waves 1 and 2 were included as research participants, which resulted in a total of 478 participants, of which 228 were male (49.1%), 236 were female (50.9%), and 14 did not fill in a gender (2.9%). There were 171 participants in grade 7 (49.1%), 85 in grade 8 (17.8%), 98 in grade 10 (20.5%), and 124 in grade 11 (25.9%), and the average age of participants was 14.04 years (SD = 1.77). 

### 2.2. Measure

Psychological *suzhi* questionnaire for middle school students (simplified version). Hu et al. revised and simplified the original Psychological *Suzhi* Questionnaire of Middle School Students developed by Yang and Zhang (the Chinese version) [40]. The questionnaire comprises 24 items and includes three dimensions: cognition quality (eight items), individuality (eight items), and adaptability (eight items). Responses for all items were made on a 5-point Likert scale ranging from 1 to 5, which reflected ‘totally disagree’ to ‘totally agree,’ respectively. The total score for all items was the final score for psychological *Suzhi,* where the higher the score, the better the psychological *suzhi*. Studies have shown this questionnaire had good reliability and validity [13,17]. In this study, Cronbach’s α of the complete questionnaire and sub-questionnaires were 0.971, 0.930, 0.924, and 0.918, respectively.

Forgiveness questionnaire for adolescents. Zhou et al. developed the Forgiveness Questionnaire for Adolescents based on Chinese culture [31]. The questionnaire consists of eight items and is divided into two dimensions: shallow and deep forgiveness. Participants were asked to respond from 1 to 6, which signified ‘completely disagree’ to ‘completely agree,’ respectively, where the higher the score, the higher the level of forgiveness. The questionnaire has shown to be an effective tool for measuring forgiveness levels in adolescents [31]. In this study, Cronbach’s α of the full questionnaire and the two dimensions were 0.889, 0.838, and 0.780, respectively.

Adolescent prosocial tendency scale. The scale to measure adolescent prosocial behavior was originally developed by Carlo and Randall, then developed into a Chinese version by Kou et al. [41,42]. It comprises 23 items and includes six dimensions: emotionality, compliance, altruism, anonymity, openness, and urgency. Responses are made on a scale from 1 to 5, which indicate ‘very unlike me’ to ‘very like me,’ respectively. Previous studies have shown that this scale is suitable for Chinese adolescents and has good reliability and validity [42]. In this study, Cronbach’s α of the full scale and subscales were 0.947, 0.759, 0.774, 0.775, 0.791, 0.830, and 0.693, respectively.

### 2.3. Procedure

This study was approved by the Ethics Committee of Jimei University. Schools and classes for this survey were selected by contacting the principals and head teachers of eligible schools. The class teachers and our researchers read out the purpose of the survey to students and informed them of their rights, which included withdrawing from participation. Participants took part in this research voluntarily, and the survey was distributed to participants at the appointed time. Then, psychology graduate students with extensive survey experience were recruited for a fee as surveyors. Before the formal investigation, our researchers trained them on preventive measures for this survey, such as controlling answer time, maintaining order, and answering students’ questions. After the data was collected, SPSS21.0 and Mplus7.0 (Created by Nie, Hull and Bent, Chicago, IL, USA, see https://www.ibm.com/products/spss-statistics for downloads) were used to complete the relevant statistical analysis work. It was necessary to have a preliminary understanding of the relationship between psychological *suzhi*, forgiveness, and adolescent prosocial behavior in the two waves. To this end, we conducted a descriptive analysis of the main variables. To determine the immediate and continuous effects of psychological *suzhi* on prosocial behavior and the mediating role of forgiveness, we constructed a structural equation model (SEM) to test the proposed mediating models twice.

## 3. Results

### 3.1. Descriptive Analysis of the Main Variables

Results showed that in wave 1, there were significant positive correlations between psychological *suzhi*, forgiveness, and prosocial behavior (*r* = 0.28–0.42 and *p* < 0.01): Psychological *suzhi* in wave1 was significantly positively associated with both forgiveness and prosocial behavior in wave 2 (*r* = 0.30, *p* < 0.01; *r* = 0.34, and *p* < 0.01), and forgiveness was significantly correlated with prosocial behavior in wave 2 (*r* = 0.50 and *p* < 0.01). In other words, regardless of the wave, psychological *suzhi* and forgiveness were closely related to the prosocial behavior of Chinese adolescents. See Table 1 for details.

### 3.2. Immediate Effect of Psychological suzhi on Prosocial Behavior

The SEM was constructed to verify the mediating role of forgiveness between psychological *suzhi* and adolescent prosocial behavior in wave 1. The model fit was accepted: χ^2^ = 361.11, *df* = 61, *p* < 0.05, CFI = 0.93, TLI = 0.91, and SRMR = 0.04. After controlling for gender and grade, the results showed that psychological *suzhi* and forgiveness significantly predicted adolescent prosocial behavior (β = 0.23, *p* < 0.001; β = 0.41, and *p* < 0.001), and psychological *suzhi* significantly predicted forgiveness (β = 0.31 and *p* < 0.001). Psychological *suzhi* and forgiveness were effective predictors of adolescent prosocial behavior, where higher levels increased prosocial behavior. Moreover, psychological *suzhi* effectively promoted improvement in forgiveness levels. For the indirect effects, the mediating effect of forgiveness between psychological *suzhi* and prosocial behavior was significant (β = 0.04; a 95% confidence interval: 0.06 and 0.20). In addition to the direct effect, psychological *suzhi* indirectly affected the current prosocial behavior of Chinese adolescents through forgiveness. See Table 2 for details.

### 3.3. Continuous Effect of Psychological suzhi on Prosocial Behavior

**Figure 1:**
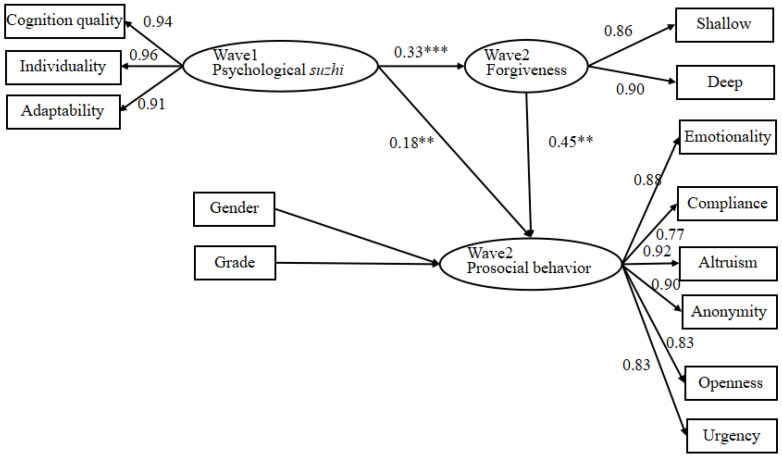
Structural Equation Model of the Continuous Effect of Psychological Suzhi on Prosocial Behavior in Wave 2 ( ** *p* < 0.01, *** *p* < 0.001).

The sustained effect of psychological *suzhi* required further verification. Here, psychological in wave 1 *suzhi* was the independent variable, forgiveness in wave 2 was the mediating variable, prosocial behavior in wave 2 was the dependent variable, and gender and grade were (CFI = 0.94, TLI = 0.92, and SRMR = 0.05). Psychological *suzhi* in wave 1 had a significant effect on both forgiveness and prosocial behavior in wave 2 (β = 0.33, *p* < 0.001; β = 0.18 and *p* < 0.01), and forgiveness had a significant impact on prosocial behavior in wave 2 (β = 0.45 and *p* < 0.001). Higher psychological *suzhi* may arouse subsequent forgiveness and increase subsequent prosocial behavior. The mediating effect of forgiveness in wave 2 on the relationship between psychological *suzhi* in wave 1and prosocial behavior in wave 2 was significant (β = 0.05, a 95% confidence interval: 0.07 and 0.22). Psychological *suzhi* had a continuous influence on adolescent prosocial behavior, and this effect was achieved indirectly through forgiveness. That is, adolescents with higher psychological *suzhi* tended to forgive others in the future and show more prosocial behaviors. See Table 2 and Figure 1 for details. 

## 4. Discussion

### 4.1. Main Findings, Comparisons, and Explanations in this Study

This study aimed to understand the mechanisms of adolescent prosocial behavior in the context of the Chinese cultural background. By investigating the Chinese psychological concept, this paper initially revealed the immediate and continuous influence of psychological *suzhi* on adolescent prosocial behavior. It demonstrated the mediating role of forgiveness on these two influences. The main findings of this study are explained below and compared with previous studies. The limitations of this research and the theoretical and practical significance of these findings are discussed. 

This study found that current psychological *suzhi* predicted not only current prosocial behavior but also subsequent prosocial behavior, which suggested that the influence of psychological *suzhi* on adolescent prosocial behavior is lasting. When psychological *suzhi* is maintained at a higher level, prosocial behaviors are more likely to occur, regardless of time. This supported hypothesis 1 of this study and is consistent with the findings of Pan et al., which revealed the continuous influence of psychological *suzhi* on the behavioral development of Chinese adolescents [11]. However, this study also complimented the baseline data, namely, the immediate impact of psychological *suzhi* on prosocial behavior, which explains the specific effect of psychological *suzhi* on the behavioral development of adolescents. The immediate and continuous effects observed in the current study are also in line with the stability feature of psychological *suzhi*, which provides empirical evidence supporting contemporary theory. In addition, positive psychology focuses on the positive psychological factors and well-being of humans. The long-term influence of psychological *suzhi* on positive behaviors also verifies the importance and indispensability of positive psychological *suzhi*. 

In this study, psychological *suzhi* had both direct and indirect effects on prosocial behavior in both waves. Adolescents with high psychological *suzhi* are more likely to have prosocial behaviors. Furthermore, adolescents with high psychological *suzhi* are more likely to forgive others and display more prosocial behaviors. In comparison to the findings of Pan et al., this study revealed different mechanisms of the effect of psychological *suzhi* on prosocial behavior. There were not only direct effects but also indirect effects of psychological *suzhi* on the prosocial behavior of adolescents in China, which demonstrates that psychological *suzhi*, an internal and stable psychological quality, can indeed affect individual behaviors without status factors. The identification of these indirect variables (the state factors) provides further options for subsequent interventions. 

This study found that forgiveness plays a mediating role in both the immediate and sustained effects of psychological *suzhi* on prosocial behavior, which confirms hypothesis 2. Psychological *suzhi* first affects forgiveness in adolescents’, subsequently, prosocial behavior. Teenagers with high psychological *suzhi* are more willing to let go of the faults of others and care less about offenses committed by others; therefore, developing close relationships and helping others is more accessible in these individuals, regardless of whether an individual is or is not an offender. A recent study in samples from the United States and Japan found that “forgiveness can be usefully conceptualized as prosocial change along a single attitudinal continuum that ranges from hostility to friendliness” [43]. Consistent with this finding, the mediating role of forgiveness in the current study also illustrated its prosocial transformation. Although forgiveness is usually regarded as a feeling in the context of Chinese culture, such a definition is not comprehensive. Instead, forgiveness is a more dynamic, rather than a static, process. 

### 4.2. Limitation and Contribution

This study explored the potential mechanisms underlying the effect of psychological *suzhi* on prosocial behavior among Chinese adolescents using a longitudinal survey. Findings in this study were supported by both baseline and longitudinal data. Therefore, the immediate and lasting influences of psychological *suzhi* on prosocial behavior were confirmed. Moreover, this study demonstrated the crucial mediating role of forgiveness. The influence of psychological *suzhi* on prosocial behavior also showed an indirect path that cannot be ignored. However, there is no doubt that this study has limitations. First, this research involved Chinese adolescents from only two waves within a semester. Although the continuous effect of psychological *suzhi* on prosocial behavior was revealed initially, this effect might be longer or even more prolonged. Second, the sample size was small, and a larger sample may allow for more profound findings. Finally, there was no international comparison. Wu et al. recently conducted a comparative study on psychological *suzhi* among Chinese and German adolescents and found that the psychological *suzhi* questionnaire of middle school students (a simplified version) was effective for both Chinese and German adolescents, showing a measurement equivalence [44]. Therefore, follow-up research should be conducted to investigate cross-cultural issues. 

However, this study investigated the occurrence of prosocial behaviors among Chinese adolescents using two waves of questionnaires, and the findings in this study confirmed the effect of psychological *suzhi* on prosocial behavior and revealed the underlying mechanism of the effect of psychological *suzhi* on prosocial behavior. This not only validates and extends the findings of Pan et al. but also enriches the field of positive psychology in regard to the effect of psychological *suzhi* on the development of human health behaviors. The mediating role of forgiveness also suggests that future research should focus on the potential role of this important variable in the development of human behavior.

### 4.3. Practical Suggestion

The general public, educators, and researchers expect individuals to exhibit as many prosocial behaviors as possible during social development. This is because most societies desire to live in a world of happiness and mutual aid. However, the implementation of positive social behavior is not an unconditional reflection but, in fact, conditional. This research focused more on individual factors rather than environmental factors and revealed the vital role of psychological *suzhi* and forgiveness in the development of prosocial behavior in Chinese adolescents. Our findings provide a helpful guide for public and educational departments and schools to implement interventions that promote adolescent prosocial behavior. On the one hand, students may undergo mental *suzhi* training as early as possible (for details, refer to Zhang’s suggestion in the book, which introduces the psychology *suzhi* training model in detail) to ensure that their mental *suzhi* is at a medium or high level [19]; on the other hand, formulating forgiveness training plans in adolescents will also be essential. Improving adolescents’ level of forgiveness is an important prerequisite for promoting prosocial behavior. 

## 5. Conclusions

This study aimed to reveal the mechanism underlying the effect of psychological *suzhi* on adolescent prosocial behavior. By analyzing two waves of data, we found that psychological *suzhi* and forgiveness were inseparable from the prosocial behavior of Chinese adolescents. Psychological *suzhi* predicted both current and subsequent prosocial behavior. Moreover, psychological *suzhi* had direct and indirect effects on adolescent prosocial behavior. In the indirect path, forgiveness played an important mediating role, regardless of whether there was an immediate or continuous influence of psychological *suzhi* on prosocial behavior. 

## Figures and Tables

**Table 1:** Descriptive Statistical Analysis Among Main Variables (N = 477).

	1	2	3	4	5
Wave1 Psychological *suzhi*	1				
Wave1 Forgiveness	0.28 **	1			
Wave1 Prosocial behavior	0.35 **	0.42 **	1		
Wave2 Forgiveness	0.30 **	0.45 **	0.31 **	1	
Wave2 Prosocial behavior	0.34 **	0.35 **	0.57 **	0.50 **	1
M	86.07	31.77	73.58	31.51	73.25
SD	18.17	7.47	16.37	7.56	17.46

Notes. ** *p* < 0.01.

**Table 2:** The Direct and Indirect Effects of Psychological Suzhi on Prosocial Behavior in Waves 1 and 2.

Paths	Effect	CI 95%
Wave 1		
Wave1 Psychological *suzhi*→Wave1 Prosocial behavior	0.23 ***	
Wave1 Psychological *suzhi*→Wave1 Forgiveness → Wave1 Prosocial behavior	0.04	[0.06, 0.20]
Wave 2		
Wave1 Psychological *suzhi*→Wave2 Prosocial behavior	0.18 **	
Wave1 Psychological *suzhi*→Wave2 Forgiveness → Wave2 Prosocial behavior	0.05	[0.07, 0.22]

Notes. ** *p* < 0.01, *** *p* < 0.001.

## Data Availability

The data is kept confidential for the time being, and the authors can be contacted if necessary.
